# Axillary lymph node metastasis in pure mucinous carcinoma of breast: clinicopathologic and ultrasonographic features

**DOI:** 10.1186/s12880-024-01290-9

**Published:** 2024-05-14

**Authors:** Na Li, Jia-Wei Li, Yu Qian, Ya-Jing Liu, Xiu-Zhu Qi, Ya-Ling Chen, Yi Gao, Cai Chang

**Affiliations:** 1 Department of Medical Ultrasound, Fudan University Shanghai Cancer Center, Shanghai, 200032 China; 2 Department of Oncology, Shanghai Medical College, Fudan University, Shanghai, 200032 China

**Keywords:** Breast neoplasms, Pure mucinous carcinoma, Axillary lymph node metastasis, Ultrasonography

## Abstract

**Background:**

The purpose of this research is to study the sonographic and clinicopathologic characteristics that associate with axillary lymph node metastasis (ALNM) for pure mucinous carcinoma of breast (PMBC).

**Methods:**

A total of 176 patients diagnosed as PMBC after surgery were included. According to the status of axillary lymph nodes, all patients were classified into ALNM group (*n* = 15) and non-ALNM group (*n* = 161). The clinical factors (patient age, tumor size, location), molecular biomarkers (ER, PR, HER2 and Ki-67) and sonographic features (shape, orientation, margin, echo pattern, posterior acoustic pattern and vascularity) between two groups were analyzed to unclose the clinicopathologic and ultrasonographic characteristics in PMBC with ALNM.

**Results:**

The incidence of axillary lymph node metastasis was 8.5% in this study. Tumors located in the outer side of the breast (upper outer quadrant and lower outer quadrant) were more likely to have lymphatic metastasis, and the difference between the two group was significantly (86.7% vs. 60.3%, *P* = 0.043). ALNM not associated with age (*P* = 0.437). Although tumor size not associated with ALNM(*P* = 0.418), the tumor size in ALNM group (32.3 ± 32.7 mm) was bigger than non-ALNM group (25.2 ± 12.8 mm). All the tumors expressed progesterone receptor (PR) positively, and 90% of all expressed estrogen receptor (ER) positively, human epidermal growth factor receptor 2 (HER2) were positive in two cases of non-ALNM group. Ki-67 high expression was observed in 36 tumors in our study (20.5%), and it was higher in ALNM group than non-ALNM group (33.3% vs. 19.3%), but the difference wasn’t significantly (*P* = 0.338).

**Conclusions:**

Tumor location is a significant factor for ALNM in PMBC. Outer side location is more easily for ALNM. With the bigger size and/or Ki-67 higher expression status, the lymphatic metastasis seems more likely to present.

## Background

Mucinous breast carcinoma (MBC) is a rare histologic type accounting for 1–7% of all breast carcinomas. According to histological characteristics, MBC can be divided into pure mucinous breast carcinoma (PMBC) without other malignant components and mixed mucinous breast carcinoma (MMBC) with non-mucinous components [1]. MMBC often contains areas of infiltrating ductal carcinoma (IDC) that are not surrounded by extracellular mucin, the imaging features and prognosis of MMBC is similar to that for IDC. PMBC consists aggregates of tumor cells surrounded by abundant pools of extracellular mucin. For this reason, PMBC usually grows more slowly and has a better prognosis [2–4]. PMBC is reported to have a low incidence of axillary lymph node metastasis (ALNM) as compared to MMBC, and the incidence of ALNM is 0–14% in PMBC [5, 6]. Axillary nodal involvement, although rare, was the most significant independent factor for the prognosis of PMBC [7, 8].

There are lots of studies focusing on risk factors for ALNM and tumor biological property in invasive breast cancers. Is that appropriate for the PMBC? To the best of our knowledge, only limited information was available concerning ALNM of PMBC because of its low incidence rate. In the present study, we investigated the association between sonographic/clinicopathological features and the ALNM in PMBC. Especially, we researched whether Ki-67 status is associated with the ALNM of PMBC, which was not mentioned in previous reports.

## Patients and methods

### Patients

With an intelligent search from the pathology records between January 2011 and January 2021, a total of 192 female patients who were diagnosed as PMBC after the primary surgery were found at Fudan University Shanghai Cancer Center. All patients accepted ultrasound examinations for bilateral breast and axilla before the surgery. Patients with the following conditions were excluded: no stored images, coexisting other types of breast cancer, with breast cancer history and multiple tumors. Finally, 176 female PMBC patients were included for the assessment of clinicopathological data as well as ultrasound images and reports. Except for suspected axillary lymph node metastasis, imaging examinations showed no other areas lymph node metastasis or distant metastasis before surgery. According to the axillary lymph node metastasis or not, the patients were divided into two groups: ALNM group and non-ALNM group. The involvement of axillary lymph nodes was ascertained by intraoperative sentinel lymph node biopsy (SLNB) and postoperative paraffin pathologic analyses after axillary lymph node dissection (ALND). This retrospective study was conducted following the ethical principles of the Declaration of Helsinki and was approved by the Ethics Committee of Fudan University Shanghai Cancer Center (registration number: 2001213-2-NSFC).

### Histopathology and immunohistochemistry analysis

All PMBC specimens obtained after surgery were routinely fixed in formalin, embedded with paraffin, and then subjected to hematoxylin-eosin staining. The microscopic slices were checked by two dedicated pathologists. The diagnosis of PMBC was ascertained when every cluster of tumor cell was partially or completely embedded within the extracellular mucus and the tumor displayed no other type of invasive carcinoma [9, 10].

Immunohistochemical status of each tumor sample was defined through immunohistochemical staining, including estrogen receptor (ER), progesterone receptor (PR), human epidermal growth factor receptor 2 (HER 2) and Ki-67. ER, PR and Ki-67 expression were defined positive if ≥ 1% nuclei staining was seen. HER-2 status was classified as negative (0/1+), positive (3+) and borderline (2+). In tumor with 2 + scores, fluorescence in situ hybridization (FISH) was performed to make a final determination. Ki-67 status was classified to low expression (<20%) and high expression (≥ 20%) .

### Assessment for ultrasonographic features

Ultrasound images of PMBC patients were collected from the data backup server and were reviewed by two senior ultrasound physicians based on the Breast Imaging Reporting and Data System (BI-RADS) lexicon [11].

The two-dimensional ultrasonographic features included:

- tumor size (the maximum diameter).
- location (outer side including the lower outer quadrant and upper outer quadrant; and inner side including the upper inner quadrant and lower inner quadrant).
- orientation (parallel and not parallel).
- shape (regular, irregular).
- margin (well-defined and ill-defined).
- echo pattern (solid echo, complex cystic and solid echo).
- posterior acoustic echo (shadow or no change, enhancement).

The vascularity of breast lesion was also retrospectively reviewed. Tumor blood flow was divided into four grades based on Adler et al. [12]: 0: no blood flow; I: small amounts of flow (one or two punctate or short rod-like color flow signals); II: medium amounts of flow (three or four punctate color flow signals or a longer blood vessel which may be half of the mass dimension long); III: rich flow (more than four punctate color flow signals or two longer blood vessels).

During all assessments, the two examiners were mutually blinded to each other as well as the patients’ clinicopathological data. Any discrepancies were resolved in consensus.

### Statistical analysis

SPSS for Windows version 22.0 (SPSS Inc., Chicago IL, USA) was used for statistical analyses. Continuous numerical data were presented as mean ± standard deviation (SD); while categorical data were presented as frequency (percentage, %). Independent samples t-test was used to compare continuous variables. Pearson’s chi-square test or Fisher’s exact test was used for comparing categorical data. All tests were two-sided. Statistical significance was defined as the two-tailed *P* value less than 0.05.

## Results

### Demographics and clinicopathological data

The demographics, clinical and histopathological characteristics of all PMBC patients were summarized in Table 1. The mean age at diagnosis was 56.7 years (range 28 ~ 95). Sixty-one patients (34.7%) were pre-menopausal and 115 (65.3%) patients were post-menopausal. ALNM not associated with age (*P* = 0.437). There were 15 patients with positive axillary lymph nodes while the other 161 patients’ axillary lymph node were negative. The incidence of ALNM was 8.5%. All the PMBCs were positive for ER expression, and 90.9% of all were positive for PR expression. Only 2 cases (1.1%) showed HER2 positivity in all PMBC patients. Ki-67 were high expressed in 36 tumors (20.5%), and it was higher in the ALNM group than non-ALNM group (33.3% vs. 19.3%), although the difference wasn’t significantly (*P* = 0.338).

**Table 1:** Clinical and histopathological characteristics of the PMBC with and without ALMN

Variables	All (*n* = 176)	ALNM (*n* = 15)	Non-ALNM (*n* = 161)	t/χ^2^	*P* value
Age (y)	56.7 ± 14.0	54.0 ± 14.0	57.0 ± 14.1	0.779	0.437
Menopausal status				1.044	0.307
Post-menopausal	115(65.3%)	8(53.3%)	107(66.5%)		
Premenopausal	61(34.7%)	7(46.7%)	54(33.5%)		
ER				-	-
Positive	176(100%)	15(100%)	161(100%)		
Negative	0(0)	0(0)	0(0)		
PR				0.357	0.898
Positive	160(90.9%)	13(86.7%)	147(91.3%)		
Negative	16(9.1%)	2(13.3%)	14(8.7%)		
HER-2				0.188	1.000
Positive	2(1.1%)	0(0)	2(1.2%)		
Negative	174(98.9%)	15(100%)	159(98.8%)		
Ki-67 level				0.918	0.338
Low(<20%)	140(79.5%)	10(66.7%)	130(80.7%)		
High (≥ 20%)	36(20.5%)	5(33.3%)	31(19.3%)		

ALNM = axillary lymph node metastasis; non-ALNM = no axillary lymph node metastasis; ER = estrogen receptor; PR = progesterone receptor; HER-2 = human epidermal growth factor receptor 2

### Ultrasonographic features of PMBC categorized by ALNM

**Fig. 1:**
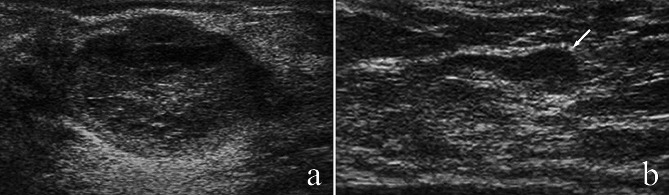
Ultrasonography of a PMBC tumor with ALNM. **(a)** Ultrasonogram shows a heterogeneous tumor with irregular shape, circumscribed margin and posterior enhancement. **(b)** Metastatic lymph nodes in the armpit with heterogeneous thickening of cortex (white arrow)

**Fig. 2:**
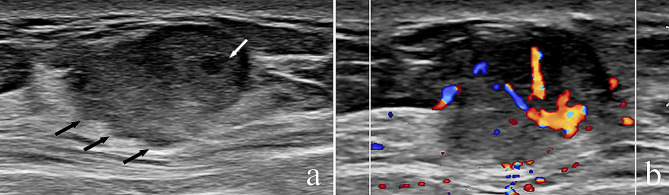
Ultrasonography of a PMBC tumor without ALNM. **(a)** Ultrasonogram shows a tumor with microlobulated margin (black arrow), complex cystic (white arrow) and solid echo. **(b)** Color Doppler flow imaging shows high level of blood flow in the tumor

Sonographic features of the PMBC with and without ALNM were shown in Table 2. Tumors located in the outer side of the breast (upper outer quadrant and lower outer quadrant) were more likely to have lymphatic metastasis, and the difference between the two group was significantly (86.7% vs. 60.3%, *P* = 0.043). The average tumor size was 25.8 mm (range 8 ~ 146 mm) in all cases. Although tumor size not associated with ALNM(*P* = 0.418), the tumor size in ALNM group (32.3 ± 32.7 mm) was bigger than non-ALNM group (25.2 ± 12.8 mm). In sonogram, most tumors present as irregular shape (89.2%), parallel orientation (83.0%), not-circumscribed margin (57.4%) and posterior acoustic enhancement (65.9%) (Fig. 1a and b). Complex cystic and solid echo pattern was present in 44 PMBC patients (25%) (Fig. 2a). Cystic component was existed in 40% of PMBC patients with ALNM, which was higher than that with no ALNM (23.6%). Tumor blood flow was grade II-III in most PMBC tumors (68.2%) (Fig. 2b). However, there is no association between these ultrasound parameters and axillary lymph node metastasis in PMBC (*P* = 0.917 for tumor shape, *P* = 0.140 for orientation, *P* = 0.447 for margin, *P* = 0.229 for posterior acoustic pattern, *P* = 0.275 for echo pattern and *P* = 0.874 for blood flow level).

**Table 2:** Ultrasonographic features of the PMBC with and without ALNM

Variables	All(*n* = 176)		ALNM (*n* = 15)	Non-ALNM (*n* = 161)	t/χ^2^	*P*-value
Size (mm)		25.8 ± 15.4	32.3 ± 32.7	25.2 ± 12.8	0.835	0.418
Location					4.086	0.043
Outer side		110(62.5%)	13(86.7%)	97(60.2%)		
Inner side		66(37.5%)	2(13.3%)	64(39.8%)		
Orientation					2.180	0.140
Parallel		146(83%)	15(100%)	131(81.4%)		
No parallel		30(17%)	0(0)	30(18.6%)		
Shape					0.290	0.917
Regular		19(10.8%)	1(6.7%)	18(11.2%)		
Irregular		157(89.2%)	14(93.3%)	143(88.8%)		
Margin					0.577	0.447
circumscribed		75(42.6%)	5(33.3%)	70(43.5%)		
Not-circumscribed		101(57.4%)	10(66.7%)	91(56.5%)		
Echo pattern					1.190	0.275
Solid echo		132(75%)	9(60%)	123(76.4%)		
Complex cystic and solid echo		44(25%)	6(40%)	38(23.6%)		
Posterior echoic					1.449	0.229
Shadowing/Normal		60(34.1%)	3(20%)	57(35.4%)		
Enhancement		116(65.9%)	12(80%)	104(64.6%)		
Blood flow level					0.201	0.874
0-I		56(31.8%)	4(26.7%)	52(32.3%)		
II-III		120(68.2%)	11(73.3%)	109(67.7%)		

ALNM = axillary lymph node metastasis; non-ALNM = no axillary lymph node metastasis; Outer side = lower outer quadrant and upper outer quadrant; Inner side = upper inner quadrant and lower inner quadrant

## Discussion

PMBC is a rare type of breast malignancy and usually has an excellent overall prognosis. However, some analysis in recent years suggest that PMBC may not be a homogeneous tumor. On some occasions, PMBC pursues an aggressive clinical course, and lymph node metastasis or even distant organ metastasis can present [5, 13].

Lymph node status, as including in the TNM stage assessment, is important for surgery type choice, systemic therapy, and assessing long-term prognosis in the common breast carcinoma [14, 15]. Axillary lymph node is the first site for lymph node metastasis, and most lymphatic vessels in the breast are draining to the axilla [16]. Although some previous studies declared that axillary lymph node staging in PMBC patients may not be necessary because PMBC seems unlikely to metastasize [17]. It is speculated that the large amount of extracellular mucus has a defensive barrier effect on infiltration, and the mucus also hinders cancer cells metabolism, thereby reducing invasive biological behavior. More and more studies with large cohorts reported that lymph node metastasis is the most significant independent prognostic factor for PMBC. Previous studies have shown the nodal status is the strongest predictor of disease-specific survival and the majority of patients presenting with axillary metastases proceeded to develop distant metastases [18, 19]. Saverio et al. [7] revealed a worse survival rate with a statistically highly significant difference between the node negative and node positive cases, and the difference was shown to be higher with longer follow up.

Compared with the common invasive ductal carcinoma of breast, PMBC is identified more frequently in elderly and post-menopausal women. In our series, 65.3% of the all patients were post-menopausal women. Lymph node metastasis is not correlation with the patients’ age as what happened in common breast carcinoma, although patient age may be a significant prognostic factor for survival [7].

Tumor size, which represents the T of TNM stage for breast cancer, is an important parameter used to evaluate the patients’ prognosis and select suitable therapy. In the previous studies for common breast carcinoma, tumor size was documented as one of the most common predictors for axillary lymph node metastasis, and there had strictly linear relationship between them [5, 20]. When the tumor is larger, with an increased likelihood of infiltration into the surrounding lymphatic vessels, which considerably increases the risk of ALNM [21]. For PMBC, some authors found that the incidence of node positivity was directly related to tumor size [7, 22], other studies did not find any correlation between tumor size and the incidence of axillary nodal metastases [17, 18, 23]. In our PMBC series, although there has a trend that tumor size in the ALNM group seems larger than that in no-ALNM group, it is not correlated with ALNM. This may be caused by the large amount of mucus which dilutes rumor cells, and the biological significance of size may mot equivalent to that of fibrous stromal tumors.

Some previous studies found common breast tumors with lateral-location were higher frequency of ALNM than medial-location ones [24, 25]. Our study showed that PMBC location in the outer side of breast (upper outer quadrants and lower outer quadrants) is more likely with positive axillary lymph nodes, which is according with common breast tumor. This finding accordance with lymphatic drainage pathway of the breast: most of the lymph fluid of the outer side of the breast is drained through the lymphatic vessels of the pectoralis lateral margin major to the axillary lymph nodes, while part of the inner side of the breast lymph fluid flows through intercostal lymphatic vessels to parasternal lymph nodes.

In our study, all the PMBC are ER+, 90.9% of all are PR + and 2 patients (1.14%) in all are Her2-positivity, which are accordance with previous studies. Ki-67 were low expressed in major PMBCs, and only 20.5% of our series expressed high level. The Ki-67 protein is a nonhistone nuclear protein encoded by the MKI-67 gene, and is exclusively related to cell proliferation [26]. Ki-67 is a well-established biomarker closely related to the development, metastasis, and prognosis of various tumors. Relevant research has shown that Ki-67 is an independent prognostic factor for breast carcinoma, and overexpression is associated with a poor prognosis for patient in the early stage [27–30]. For PMBCs, Pan et al. reported that high Ki-67 appeared as disease-free survival factor [5]. Moreover, many researchers have documented that Ki-67 expression is associated with lymph node metastasis in common breast carcinoma [31–33]. In our PMBC series, 20.5% of all PMBC were documented with high expression of Ki-67, while in ALNM and non-ALNM group it was 33.3% and 19.3% respectively. Although Ki-67 not associated with ALNM, there was a trend that the ALNM group had a higher Ki-67 express.

Compared with common invasive ductal carcinoma of breast, there have some obvious differences in sonographic features of PMBC, such as circumscribed margin, regular shape and enhancement posterior echoic. Previous studies reported that some sonographic features, such as tumor shape, margin, internal echo and vascularity, were significantly associated with ALNM [33, 34]. But the pathological type collected in previous studies were major in common invasive ductal carcinoma. For our PMBC series, they all not associated with ALNM. Conventional ultrasound image seems limit in distinguishing PMBC axillary lymph node metastasis trending.

The limitations of this study should be mentioned. Firstly, this is a single-center analysis and the sample size is relatively small, especially for patients with ALNM. The bias is inevitable for this study due to the low incidence rate of ALNM in PMBC cohorts. Further studies with larger cohorts are warranted. Secondly, this is a retrospective analysis of still US images which may cause missing or misinterpreting information. Thirdly, the ultrasound parameters in this study are only obtained from conventional ultrasound. Some new technology, like radiomics and ultrasonic radio frequency signal analysis, will be performed to explore more.

## Conclusion

Tumor location is a significant factor for axillary lymphatic metastasis in PMBC. Outer side location is more easily for axillary lymph node metastasis. With the bigger size and Ki-67 higher expression status, the lymphatic metastasis seems more likely to present. Unlike invasive ductal carcinoma, there is no association between conventional ultrasound image parameters and axillary lymph node metastasis in PMBC. More samples or new ultrasonic technology, like radiomics and ultrasonic radio frequency signal analysis, should be performed to explore more.

## Data Availability

The datasets used and analyzed during this study are available from the corresponding author on reasonable request.

## Abbreviations

PMBC: Pure mucinous of breast carcinoma
MMBC: Mucinous breast carcinoma
MBC: Mucinous breast carcinoma
ALNM: Axillary lymph node metastasis
non-ALNM: no axillary lymph node metastasis
ER: Estrogen receptor
PR: Progesterone receptor
HER-2: Human epidermal growth factor receptor 2
IDC: Invasive ductal carcinoma
SLNB: Sentinel lymph node biopsy
ALND: Axillary lymph node dissection

## References

[1] Lakhani SR, Ellis IO, Schnitt SJ, Tan PH, van de Vijver MJ (2012). WHO classification of tumours of the breast.

[2] Hashmi AA, Zia S, Yaqeen SR, Ahmed O, Asghar IA, Islam S (2021). Mucinous breast carcinoma: clinicopathological comparison with invasive ductal carcinoma. Cureus.

[3] Marrazzo E, Frusone F, Milana F, Sagona A, Gatzemeier W, Barbieri E (2020). Mucinous breast cancer: a narrative review of the literature and a retrospective tertiary single-centre analysis. Breast.

[4] Zhou XT, Zheng ZB, Li Y, Zhao WW, Lin Y, Zhang JS (2021). The clinical features and prognosis of patients with mucinous breast carcinoma compared with those with infiltrating ductal carcinoma: a population-based study. BMC Cancer.

[5] Pan B, Yao R, Shi J, Xu QQ, Zhou YD, Mao F (2016). Prognosis of subtypes of the mucinous breast carcinoma in Chinese women: a population-based study of 32-year experience (1983–2014). Oncotarget.

[6] Lei L, Yu XF, Chen B, Chen ZH, Wang XJ (2016). Clinicopathological characteristics of mucinous breast cancer: a retrospective analysis of a 10-year study. PLoS ONE.

[7] Saverio SD, Gutierrez J, Avisar E (2008). A retrospective review with long term follow up of 11,400 cases of pure mucinous breast carcinoma. Breast Cancer Res Treat.

[8] Cao AY, He M, Liu ZB, Di GH, Wu J, Lu JS (2012). Outcome of pure mucinous breast carcinoma compared to infiltrating ductal carcinoma: a population-based study from China. Ann Surg Oncol.

[9] Kashiwagi S, Onoda N, Asano Y, Noda S, Kawajiri H, Takashima T (2013). Clinical significance of the subclassification of 71 cases mucinous breast carcinoma. Springerplus.

[10] Monzawa S, Yokokawa M, Sakuma T, Takao S, Hirokaga K, Hanioka K (2009). Mucinous carcinoma of the breast: MRI features of pure and mixed forms with histopathologic correlation. AJR Am J Roentgenol.

[11] Mendelson EB, Böhm-Vélez M, Berg WA (2013). ACR BI-RADS® Ultrasound. ACR BI-RADS® Atlas, breast imaging reporting and Data System.

[12] Adler DD, Carson PL, Rubin JM, Quinn-Reid D (1990). Doppler ultrasound color flow imaging in the study of breast cancer: preliminary findings. Ultrasound Med Biol.

[13] Ross JS, Gay LM, Nozad S, Wang K, Ali SM, Boguniewicz A (2016). Clinically advanced and metastatic pure mucinous carcinoma of the breast: a comprehensive genomic profiling study. Breast Cancer Res Treat.

[14] Williams PA, Suggs J, Mangana SH (2014). Axillary lymph node treatment in breast cancer: an update. J Miss State Med Assoc.

[15] Rao R, Euhus D, Mayo HG, Balch C (2013). Axillary node interventions in breast cancer: a systematic review. JAMA.

[16] Schwartz RS, Erban JK (2017). Timing of metastasis in breast cancer. N Engl J Med.

[17] Paramo JC, Wilson C, Velarde D, Giraldo J, Poppiti RJ, Mesko TW (2002). Pure mucinous carcinoma of the breast: is axillary staging necessary?. Ann Surg Oncol.

[18] Komenaka IK, El-Tamer MB, Troxel A, Hamele-Bena D, Joseph KA, Horowitzet E (2004). Pure mucinous cainoma of the breast. Am J Surg.

[19] Clayton F (1986). Pure mucinous carcinomas of the breast: morphologic features and prognostic correlates. Hum Pathol.

[20] Kasangian AA, Gherardi G, Biagioli E, Torri V, Moretti A, Bernardin E (2017). The prognostic role of tumor size in early breast cancer in the era of molecular biology. PLoS ONE.

[21] Barth A, Craig PH, Silverstein MJ (1997). Predictors of axillary lymph node metastases in patients with T1 breast carcinoma. Cancer.

[22] Diab SG, Clark GM, Osborne CK, Libby A, Allred DC, Elledge RM (1999). Tumor characteristics and clinical outcome of tubular and mucinous breast carcinoma. J Clin Oncol.

[23] Avisar E, Khan MA, Axelrod D, Oza K (1998). Pure mucinous carcinoma of the breast: a clinicopathologic correlation study. Ann Surg Oncol.

[24] Bevilacqua J, Rd CH, Macdonald KA, Tan LK, Borgen PI, Van Zee KJ (2002). A prospective validated model for predicting axillary node metastases based on 2,000 sentinel node procedures: the role of tumour location. Eur J Surg Oncol.

[25] Zhang YX, Li J, Fan Y, Li XM, Qiu JJ, Zhu M (2019). Risk factors for axillary lymph node metastases in clinical stage T1-2N0M0 breast cancer patients. Med (Baltim).

[26] Menon SS, Guruvayoorappan C, Sakthivel KM, Rasmi RR (2019). Ki-67 protein as a tumour proliferation marker. Clin Chim Acta.

[27] Yerushalmi R, Woods R, Ravdin PM, Hayes MM, Gelmon KA (2010). Ki67 in breast cancer: prognostic and predictive potential. Lancet Oncol.

[28] Wang W, Wu JY, Zhang PF, Fei XC, Zong Y, Chen XS (2016). Prognostic and predictive value of KI-67 in triple-negative breast cancer. Oncotarget.

[29] Yuan P, Xu BL, Wang CZ, Zhang CJ, Sun MM, Yuan L (2016). Ki-67 expression in luminal type breast cancer and its association with the clinicopathology of the cancer. Oncol Lett.

[30] Matsubara N, Mukai H, Itoh K, Nagai S (2011). Prognostic impact of Ki-67 overexpression in subgroups categorized according to St. Gallen with early stage breast cancer. Oncology.

[31] Chung MJ, Lee JH, Kim SH, Suh YJ, Choi HJ (2016). Simple prediction model of axillary lymph node positivity after analyzing molecular and clinical factors in early breast cancer. Med (Baltim).

[32] Adamo B, Ricciardi GRR, Ieni A, Franchina T, Fazzari C, Sanò MV (2017). The prognostic significance of combined androgen receptor, E-cadherin, Ki-67 and CK5/6 expression in patients with triple negative breast cancer. Oncotarget.

[33] Zhang H, Sui XF, Zhou SZ, Hu L, Huang X (2019). Correlation of conventional ultrasound characteristics of breast tumors with axillary lymph node metastasis and Ki-67 expression in patients with breast cancer. J Ultrasound Med.

[34] Guo Q, Dong ZW, Zhang L, Ning CP, Li ZY, Wang DM, et al. Ultrasound features of breast cancer for predicting axillary lymph node metastasis. J Ultrasound Med. 2018;37(6):1345–53. 10.1002/jum.14469 29119589

